# Dynamic monitoring of immune function indexes in COVID-19 patients

**DOI:** 10.18632/aging.202362

**Published:** 2020-12-23

**Authors:** Yaohui Song, Huixiu Zhong, Lin Li, Minggang Yin, Yi Yin, Xiaolong Guo, Bo Zhang, Weiping Liu

**Affiliations:** 1 Department of Clinical Laboratory, Zigong First People’s Hospital, Zigong 643000, Sichuan, China; 2 Department of Laboratory Medicine, Sichuan Academy of Medical Sciences and Sichuan Provincial People’s Hospital, Chengdu 610072, Sichuan, China

**Keywords:** COVID-19, dynamic monitoring, early diagnostic markers, IgE, SARS-CoV-2 antibodies

## Abstract

We conducted a retrospective analysis of the clinical characteristics and dynamic variations of immune indexes in nine COVID-19 patients in Zigong, China. We used flow cytometry and enzyme-linked immunosorbent assays to measure the absolute levels of CD4 and CD8 lymphocytes and SARS-CoV-2 antibodies, respectively. We found that CRP, LDH, HBDH, CD4/CD8 and IgE levels were increased in 6/9 patients, while PA and the absolute numbers of CD4 and CD8 lymphocytes decreased in 7/9 patients. From disease onset through 63 days of follow-up, SARS-CoV-2 IgG levels were consistently higher than those of SARS-CoV-2 IgM, reaching peaks on days 28 and 13, respectively. IgM levels decreased to normal 35 days after disease onset, while IgG levels remained elevated through day 63. IgE levels varied similarly to SARS-CoV-2 IgM. Our results suggest that SARS-CoV-2 may elicit allergic immune responses in patients and that the levels of CRP, PA, LDH, and HBDH, as well as the absolute numbers of CD4 and CD8 lymphocytes could be used as early diagnostic markers of SARS-CoV-2 infection. Lastly, the dynamic variation of SARS-CoV-2 antibodies could guide the timing of blood collection for plasma exchange.

## INTRODUCTION

At the end of April 2020, the spread of COVID-19 had been controlled with strong measures in China, but it had spread widely around the rest of the world [1]. Apart from supportive care and traditional Chinese medicine treatment, specific drugs and/or vaccines for COVID-19 are still under clinical research [2, 3]. Previously, plasma or immunoglobulins from patients with higher antibody levels who recovered from the infection were used to treat Severe Acute Respiratory Syndrome (SARS) patient sand were found to be a useful treatment without serious adverse events [1, 4, 5]. Therefore, it might be useful to explore whether plasma from patients who recovered from COVID-19 could be used for plasmapheresis treatment of COVID-19 patients actively battling severe infection. Here, we evaluated the dynamic variation law of immune function indexes of COVID-19 patients. In addition, we report the damage to immune functions and the recovery time for patients after SARS-CoV-2 infection. So far, several studies have described the epidemiological and clinical characteristics of patients infected with COVID-19, but there have been no reports on the dynamic monitoring of immune indexes in infected persons [3, 6, 7]. In this study, we retrospectively analyzed the clinical characteristics and laboratory tests of 9 patients in the Zigong area diagnosed with COVID-19. In addition, several differentially-expressed indicators associated with inflammation and immunity, including C-reactive protein (CRP), prealbumin (PA), absolute values of CD4 lymphocytes and CD8 lymphocytes, IgE, SARS-CoV-2 IgM and SARS-CoV-2 IgG were comprehensively analyzed to explore the inflammatory and immune response of the body to the SARS-CoV-2 virus, and to find out the dynamic variation law of SARS-CoV-2 antibodies production in COVID-19 patients. These indicators were tested from the beginning of hospitalization to 63 days after discharge of patients. The present results will provide a new basis for clinical diagnosis, treatment and prognosis of COVID-19.

## RESULTS

### Demographics, baseline and clinical characteristics of patients infected with SARS-COV-2

In this study, nine cases (seven females and two males) infected with SARS-COV-2 were investigated in Zigong City, China. All these cases were imported infections (with a history of epidemic in Wuhan). Among them, six patients were aged 30-49 years, one (case 7) was aged 20 years, and another (case 2) was aged 67 years. The demographic and clinical characteristics are shown in Table 1. Of the nine patients, four had underlying comorbidities, including syringomyelia, hypertension, fatty liver, and diabetes (Table 1). In terms of clinical classification, four patients were mild type, and five were moderate type. The most common symptoms were fever and cough, which accounted for eight cases and seven cases respectively. Four cases had shortness of breath. In addition, three patients had fatigue and chest tightness, one had myalgia and sore throat. The chest X-rays of all patients were abnormal, including bilateral lung involvement in six cases and unilateral lung involvement in three (cases 3, 4, and 8). The most common radiologic manifestations were ground-glass opacities and patchy shadows (Table 1). All patients received traditional Chinese medicine and antiviral treatment, including lopinavir/ritonavir, and five cases were treated with antibiotics. Three consecutive negative SARS-CoV-2 nucleic acid testing results using throat swab samples without fever, cough, dyspnea, shortness of breath, abdominal pain and diarrhea present were used as the standard for discharging patients, according to the guideline for the diagnosis and treatment of COVID-19 (trial version 7) released by National Health Commission of the People’s Republic of China. As a result, all patients were discharged before February 27, 2020.

**Table 1:** Demographics, baseline and clinical characteristics of patients infected with SARS-COV-2.

**Variables**	**case 1**	**case 2**	**case 3**	**case 4**	**case 5**	**case 6**	**case 7**	**case 8**	**case 9**
Age(y)	52	67	48	40	34	49	20	42	39
Sex	Male	Female	Female	Female	Male	Female	Female	Female	Female
Clinical classification	Moderate type	Moderate type	Moderate type	Mild type	Moderate type	Mild type	Mild type	Mild type	Moderate type
Comorbidities	Syringomyelia	Hypertension	Hypertension, fatty liver	diabetes	none	none	none	none	none
Signs and symptoms at admission									
Fever	Yes	Yes	Yes	Yes	Yes	Yes		Yes	Yes
Cough	Yes	Yes	Yes	Yes		Yes	Yes		
Myalgia		Yes							
Fatigue	Yes	Yes	Yes						
Sore throat		Yes							
Chest tightness	Yes	Yes	Yes						Yes
Chest x-ray and CT findings									
Unilateral pneumonia			Yes	Yes				Yes	
Bilateral pneumonia	Yes	Yes			Yes	Yes	Yes		Yes
Treatment									
Antibiotic treatment	Yes	Yes	Yes		Yes	Yes			
Antiviral treatment	Yes	Yes	Yes	Yes	Yes	Yes	Yes	Yes	Yes
Intravenous immunoglobulin therapy	Yes	Yes	Yes	Yes	Yes	Yes	Yes	Yes	Yes
Traditional Chinese medicine	Yes	Yes	Yes	Yes	Yes	Yes	Yes	Yes	Yes
Days from illness onset to hospitalization	4	1	2	2	4	2	1	1	6
Days from illness onset to discharge	29	25	23	15	31	27	16	23	26
Clinical outcome	Discharged	Discharged	Discharged	Discharged	Discharged	Discharged	Discharged	Discharged	Discharged

### Laboratory findings of patients infected with SARS-CoV-2

Before treatment, three patients showed lower white blood cell (WBC) count, three had increased neutrophil ratio and decreased lymphocyte ratio, and seven had elevated CRP (Supplementary Table 1). In terms of T cell subsets, the absolute values of CD4 lymphocytes and CD8 lymphocytes in seven patients were lower than the normal reference range. At the same time, CD4/CD8 levels were higher than the normal reference range for six patients. For immunoglobulins, IgM and IgA levels of all patients were normal, while the IgG levels of two patients were slightly lower than normal. However, IgE levels were increased in all patients. In terms of SARS-CoV-2 antibodies, only one patient had elevated levels of SARS-CoV-2 IgM, while six patients had higher SARS-CoV-2 IgG levels. In terms of liver function, six patients showed total protein below the normal range, while eight had reduced albumin level. Only one patient had increased alanine transaminase (ALT) while three had aspartate aminotransferase (AST) levels above the normal range. The levels of PA was decreased in seven patients and hydroxybutyrate dehydrogenase (HBDH) levels were increased in all patients, including seven patients with elevated lactic dehydrogenase (LDH) (Supplementary Table 1).

After treatment, the total leukocyte count, neutrophil and lymphocyte ratio, CRP, PA, ALT, AST, LDH and HBDH levels were normal in all patients. In addition, the absolute values of CD4 lymphocytes and CD8 lymphocytes in all patients reached normal levels, but there were still three patients whose CD4/CD8 levels were elevated. Surprisingly, the level of IgE in all patients after recovering from the illness was higher than that before treatment. After discharge from the hospital, two patients tested negative for SARS-CoV-2 IgM while the other seven tested positive for SARS-CoV-2 IgM and SARS-CoV-2 IgG (Supplementary Table 1).

### Changes of indexes related to inflammation and immune function in patients

**Figure 1:**
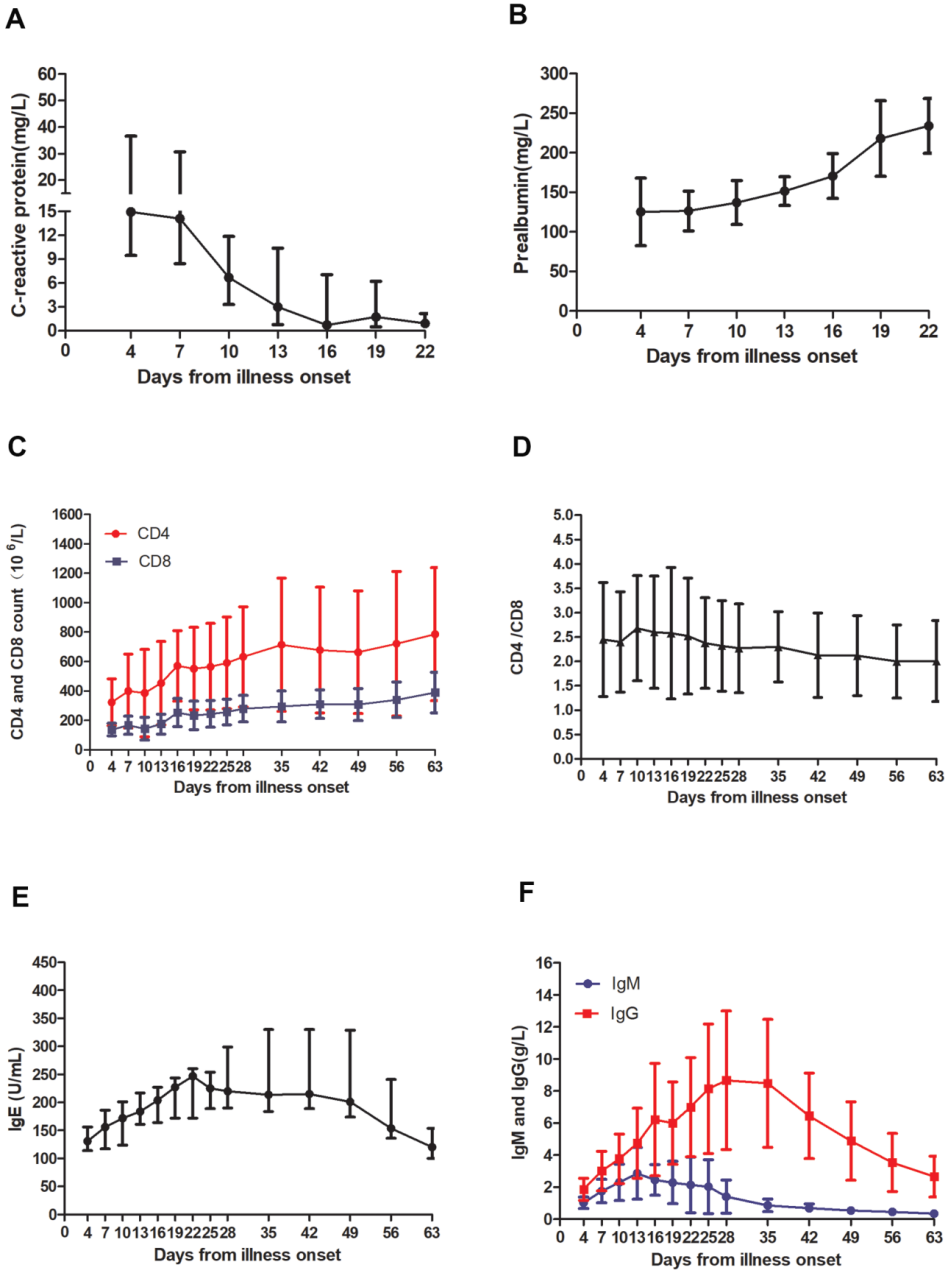
**Temporal changes in laboratory markers from illness onset in patients hospitalized with COVID-19.** Temporal changes in (**A**) C-reactive protein, (**B**) prealbumin, (**C**) the absolute values of CD4 and CD8, (**D**) CD4/CD8, (**E**) IgE, and (**F**) SARS-CoV-2 IgM and SARS-CoV-2 IgG. (**A**, **E**) the data are presented as the median (quartile) *M(P25,P25)*, (**B**, **C**, **D**, **F**) the data are displayed as the mean ± SD.

Several major laboratory markers were selected from the above laboratory results and were tracked from illness onset. The CRP curve showed increased CRP levels (14.93 mg/L (9.47 mg/L, 36.56 mg/L)) in the blood of the patients at the beginning of the disease, which dropped to the normal levels (0.75mg/L (0.60mg/L, 7.05mg/L)) after 16 days. On the contrary, through the PA generation curve, we observed that PA levels (125.33±42.62 mg/L) were decreased at early stages of the illness and return to normal (218±47.89 mg/L) after 19 days. The absolute values of CD4 and CD8 lymphocytes both decreased at the initial stage of onset, then began to rise, and returned to normal after 16 days of onset. Then it continued to rise. Followed up to 63 days, it rose to the median of the normal range. For CD4/CD8, it was higher than the normal range in the early stage (2.45±1.17), with the progression of the disease, the CD4/CD8 ratio reached the highest level on the 10^th^ day (2.68±1.08), then it began to decline, and the overall level (2.00±0.83) fell to the normal range from day 56 after disease onset. However, IgE levels (131mg/L (114mg/L, 156mg/L)) began to increase from the time of onset, and peaked (247 mg/L (172 mg/L, 260 mg/L)) on day 22 after illness onset, then began to decline. Until day 63 after the onset, IgE levels (120mg/L (100 mg/L, 154mg/L)) were reduced, but it still did not fall to the normal range (0-100). We also found that SARS-CoV-2 IgM and SARS-CoV-2 IgG gradually increased with disease progression, with greater increase for SARS-CoV-2 IgG than for SARS-CoV-2 IgM. The highest levels of SARS-CoV-2 IgM (2.86±1.61mg/L) and SARS-CoV-2 IgG (8.67±4.33mg/L) occurred on the 13^th^ and 28^th^ day of the disease, respectively, and were 2.80x and 4.69x higher than at the initial stage of the disease, respectively. On the 35^th^ day after the onset of the disease, SARS-COV-2 IgM level (0.34±0.05mg/L) decreased to normal levels, while SARS-CoV-2 IgG (2.66±1.28 mg/L) remained elevated even at day 63 after disease onset (Figure 1).

## DISCUSSION

Here, we reported 9 cases of SARS-CoV-2 infection in Zigong, China, all of whom were “imported infections”, except for two patients who lived in Wuhan for a long time. All patients received timely diagnosis and treatment and were discharged by the end of February 2020.

In terms of clinical classification, all patients had mild or moderate pneumonia, the main symptoms being fever and cough, consistent with previous reports of COVID-19 [8]. Other common symptoms of the disease are chills or headache, myalgia, fatigue and cough [9]. In this study, one patient presented elevated troponin levels and myocarditis and four had shortness of breath. In addition, three patients had fatigue and chest tightness. Except for two patients with mild SARS-CoV-2 pneumonia, all patients were treated with integrated traditional Chinese and western medicine.

In terms of laboratory tests at early infection stages, three patients had a decreased WBC, one third of the patients had an increased neutrophil ratio and decreased lymphocyte ratio, and most patients had elevated CRP levels, generally consistent with previous reports [10]. The SARS-CoV-2 virus invades the human body through the respiratory tract, causing a series of immune reactions in the body such as the induction of a cytokine storm and decreased white blood cells and lymphocytes in peripheral blood [11]. It has been reported that SARS-CoV-2 may directly bind to ACE2-positive cholangiocytes, therefore, infected patients are prone to liver dysfunction [6]. In our study, three patients had increased AST, but only one had abnormal ALT. In most patients, albumin and PA levels were decreased. Previous studies have shown that the prealbumin levels are reduced in the serum of patients with HAdV-7 infection, which can be used as an inflammatory marker of adenovirus respiratory infection [12]. However, our results here showed that LDH and HBDH levels exhibited opposite trends, consistent with previous reports of patients infected with SARS-CoV-2 [13–15]. LDH has been reported to be a risk factor for SARS-CoV-2 patients and can be used as an auxiliary marker to predict the severity of tissue damage in the early stage of the disease [14–16]. Other results from our study here showed that CRP, PA, LDH and HBDH levels returned to normal on the 19^th^ day after onset, suggesting that they can be used as early serological inflammatory markers of SARS-CoV-2 infection.

In terms of immune function, in the early stages of the disease, the absolute values of CD4 and CD8 lymphocytes in the patient's peripheral blood were decreased, while CD4/CD8 were increased. As the disease progressed, the absolute values of CD4 and CD8 lymphocytes returned to normal, but CD4/CD8 remained elevated. During the patient follow-up period, CD4/CD8 gradually decreased, reaching normal levels at day 63 after disease onset. Unlike what is seen in other viral infections, T lymphocyte subsets were reduced in patients with SARS-CoV-2 infection, which has been shown to correlate positively with hospital mortality and disease severity, suggesting that T lymphocyte levels could be used as a biomarker for early diagnosis [17–19]. Previous studies have shown that SARS-CoV-2 IgM and SARS CoV-2 IgG can be used as protective antibodies to treat patients with severe infection through plasma exchange [2]. Our detection of SARS-CoV-2 antibodies in patient sera showed that most patients produce the IgG antibody first and then the IgM antibody, with both antibodies increasing steadily until peaking on day 13 and day 28, respectively. While SARS-CoV-2 IgM levels decreased to normal by day 35, those of SARS-CoV-2 IgG remained elevated even at day 63 while IgE levels increase from the onset to day 28, after which they began to decrease but did not fall to normal levels until day 63. Therefore, we hypothesize that reducing IgE levels could be an effective treatment for IgE-mediated allergic reactions in COVID-19 patients. Recent studies have shown that blocking IgE can reduce the susceptibility to respiratory virus infections by enhancing IFN-α signal in plasmacytoid dendritic cells and that allergen-specific immunotherapy (AIT) is one of the most important treatment options for IgE-mediated allergic reactions [20–22].

In summary, our research showed that the levels of CRP, PA, LDH, and HBDH, as well as absolute values of CD4 and CD8 lymphocytes, might be used as early diagnostic markers for SARS-CoV-2 infection. In addition, through dynamic monitoring of SARS-CoV-2 IgM and SARS -CoV-2 IgG antibody levels, we measured the changes in antibody levels in infected patients, revealing the best donor blood collection time for plasma exchange treatment. At the same time, by measuring changes in IgE levels in patients, we found that SARS-CoV-2 might cause allergic immune responses. Studies with much larger numbers of clinical samples are warranted and could strengthen the exciting preliminary results of this study.

## MATERIALS AND METHODS

### Patients

We retrospectively investigated the clinical, imaging, and laboratory characteristics of diagnosed cases of SARS-CoV-2 with WHO interim guidance in Zigong First People’s Hospital, which is the only designated hospital in the city for 2019-nCoV pneumonia from Jan 16 to Jan 31, 2020. The clinical outcomes were monitored up to Apr 2th, 2020, the final date of follow-up. The study was approved by the ethic commissions of Zigong First People’s Hospital, and determined to be exempt from oversight given the use of pre-existing, de-identified data. As such, individual-level informed consent was not obtained. Eleven kinds of respiratory pathogens and influenza A and B nucleic acids were examined in all patients, and bacteria and fungi were cultured. The results showed that all patients were negative for the pathogens above.

### Data collection

Epidemiological, clinical, laboratory, management and outcome data were collected through a review of medical records. Clinical outcomes were followed up until April 2, 2020. Laboratory confirmation of SARS-CoV-2 was done in Zigong Center for Disease Control and Prevention. Throat-swab specimens from the upper respiratory tract that were obtained from all patients at admission were maintained in viral-transport medium. The presence of SARS-CoV-2 was confirmed by real-time RT-PCR. All patients were subjected to chest computed tomography (CT) or chest radiography.

### Flow cytometry and enzyme-linked immunosorbent assays

Peripheral venous blood (~2 mL) was collected from COVID-19 patients in EDTA anticoagulant tubes and ordinary biochemical tubes, respectively. The blood samples were centrifuged to obtain serum and were stored at -80° C. T lymphocyte subsets were detected immediately after EDTA anticoagulant blood samples were drawn, as described below. We took 50μL whole blood, added 5μL CD3-PC7(REF,737657), CD4-APC (REF, IM2468), CD8-ECD(REF, 6604728) and CD45-FITC(REF, 737649) monoclonal antibodies, mixed all ingredients by shaking, incubated the mixture at room temperature and avoided exposure to light for 20 min; we then added 300μL of red blood cell lysate, incubated the samples for 10 min, added 1.5 mL phosphate buffer saline (PBS) to wash the samples once, spun them at 1 000 r/min, centrifugation for 5 min, discarded the supernatant, and added 500μL PBS, before mixing again by shaking, using standard fluorescent microspheres to calibrate the instrument. Beckman Coulter cytomics^TM^ FC 500 five-color flow cytometry was used for detection and analysis. The reagents were purchased from Beckman Coulter company in the United States. The SARS-CoV-2 IgM and SARS-CoV-2 IgG were detected by ELISA. The reagents were purchased from Zhuhai Lizhu Reagent Co., Ltd. All operations were carried out in strict accordance with the instructions.

### Statistical methods

Analysis of normality and homogeneity of variance was carried out on the measurement data. Data that exhibited a normal distribution was displayed as $\bar{x}\pm s$; otherwise, it was expressed as the median (quartile) *M*(*P*25, *P*75). Other data were displayed as tables and line charts.

## Supplementary Material

Supplementary Tables

## References

[1] Chen L, Xiong J, Bao L, Shi Y. Convalescent plasma as a potential therapy for COVID-19. Lancet Infect Dis. 2020; 20:398–400. 10.1016/S1473-3099(20)30141-9 32113510 PMC7128218

[2] Zhang L, Pang R, Xue X, Bao J, Ye S, Dai Y, Zheng Y, Fu Q, Hu Z, Yi Y. anti-SARS-CoV-2 virus antibody levels in convalescent plasma of six donors who have recovered from COVID-19. Aging (Albany NY). 2020; 12:6536–42. 10.18632/aging.103102 32320384 PMC7202482

[3] Wang Z, Chen X, Lu Y, Chen F, Zhang W. Clinical characteristics and therapeutic procedure for four cases with 2019 novel coronavirus pneumonia receiving combined Chinese and Western medicine treatment. Biosci Trends. 2020; 14:64–68. 10.5582/bst.2020.01030 32037389

[4] Ko JH, Seok H, Cho SY, Ha YE, Baek JY, Kim SH, Kim YJ, Park JK, Chung CR, Kang ES, Cho D, Müller MA, Drosten C, et al. Challenges of convalescent plasma infusion therapy in Middle East respiratory coronavirus infection: a single centre experience. Antivir Ther. 2018; 23:617–22. 10.3851/IMP3243 29923831

[5] Tedder RS, Samuel D, Dicks S, Scott JT, Ijaz S, Smith CC, Adaken C, Cole C, Baker S, Edwards T, Kamara P, Kargbo O, Niazi S, et al, and Ebola_CP Consortium Investigators. Detection, characterization, and enrollment of donors of ebola convalescent plasma in Sierra Leone. Transfusion. 2018; 58:1289–98. 10.1111/trf.14580 29572862 PMC5947131

[6] Wu J, Liu J, Zhao X, Liu C, Wang W, Wang D, Xu W, Zhang C, Yu J, Jiang B, Cao H, Li L. Clinical characteristics of imported cases of coronavirus disease 2019 (COVID-19) in Jiangsu province: a multicenter descriptive study. Clin Infect Dis. 2020; 71:706–12. 10.1093/cid/ciaa199 32109279 PMC7108195

[7] Zhou F, Yu T, Du R, Fan G, Liu Y, Liu Z, Xiang J, Wang Y, Song B, Gu X, Guan L, Wei Y, Li H, et al. Clinical course and risk factors for mortality of adult inpatients with COVID-19 in Wuhan, China: a retrospective cohort study. Lancet. 2020; 395:1054–62. 10.1016/S0140-6736(20)30566-3 32171076 PMC7270627

[8] Liu K, Fang YY, Deng Y, Liu W, Wang MF, Ma JP, Xiao W, Wang YN, Zhong MH, Li CH, Li GC, Liu HG. Clinical characteristics of novel coronavirus cases in tertiary hospitals in Hubei Province. Chin Med J (Engl). 2020; 133:1025–31. 10.1097/CM9.0000000000000744 32044814 PMC7147277

[9] Xiao Y, Peng Z, Tan C, Meng X, Huang X, Wu A, Li C. Diagnostic options for coronavirus disease 2019 (COVID-19). Infect Control Hosp Epidemiol. 2020; 41:1358–59. 10.1017/ice.2020.168 32321609 PMC7303465

[10] Wang Z, Yang B, Li Q, Wen L, Zhang R. Clinical Features of 69 Cases With Coronavirus Disease 2019 in Wuhan, China. Clin Infect Dis. 2020; 71:769–77. 10.1093/cid/ciaa272 32176772 PMC7184452

[11] Song F, Shi N, Shan F, Zhang Z, Shen J, Lu H, Ling Y, Jiang Y, Shi Y. Emerging 2019 Novel Coronavirus (2019-nCoV) Pneumonia. Radiology. 2020; 295:210–17. 10.1148/radiol.2020200274 32027573 PMC7233366

[12] Sun J, Xiao Y, Zhang M, Ao T, Lang S, Wang J. Serum Inflammatory Markers in Patients with Adenovirus Respiratory Infection. Med Sci Monit. 2018; 24:3848–55. 10.12659/MSM.910692 29877315 PMC6020746

[13] Zhang Y, Zheng L, Liu L, Zhao M, Xiao J, Zhao Q. Liver impairment in COVID-19 patients: a retrospective analysis of 115 cases from a single centre in Wuhan city, China. Liver Int. 2020; 40:2095–103. 10.1111/liv.14455 32239796

[14] Gong J, Ou J, Qiu X, Jie Y, Chen Y, Yuan L, Cao J, Tan M, Xu W, Zheng F, Shi Y, Hu B. A tool for early prediction of severe coronavirus disease 2019 (COVID-19): a multicenter study using the risk nomogram in Wuhan and Guangdong, China. Clin Infect Dis. 2020; 71:833–40. 10.1093/cid/ciaa443 32296824 PMC7184338

[15] Zhao D, Yao F, Wang L, Zheng L, Gao Y, Ye J, Guo F, Zhao H, Gao R. A Comparative Study on the Clinical Features of Coronavirus 2019 (COVID-19) Pneumonia With Other Pneumonias. Clin Infect Dis. 2020; 71:756–61. 10.1093/cid/ciaa247 32161968 PMC7108162

[16] Mo P, Xing Y, Xiao Y, Deng L, Zhao Q, Wang H, Xiong Y, Cheng Z, Gao S, Liang K, Luo M, Chen T, Song S, et al. Clinical characteristics of refractory COVID-19 pneumonia in Wuhan, China. Clin Infect Dis. 2020. [Epub ahead of print]. 10.1093/cid/ciaa270 32173725 PMC7184444

[17] Xu B, Fan CY, Wang AL, Zou YL, Yu YH, He C, Xia WG, Zhang JX, Miao Q. Suppressed T cell-mediated immunity in patients with COVID-19: a clinical retrospective study in Wuhan, China. J Infect. 2020; 81:e51–60. 10.1016/j.jinf.2020.04.012 32315725 PMC7166040

[18] Zheng Y, Xu H, Yang M, Zeng Y, Chen H, Liu R, Li Q, Zhang N, Wang D. Epidemiological characteristics and clinical features of 32 critical and 67 noncritical cases of COVID-19 in Chengdu. J Clin Virol. 2020; 127:104366. 10.1016/j.jcv.2020.104366 32302954 PMC7146675

[19] Chen G, Wu D, Guo W, Cao Y, Huang D, Wang H, Wang T, Zhang X, Chen H, Yu H, Zhang X, Zhang M, Wu S, et al. Clinical and immunological features of severe and moderate coronavirus disease 2019. J Clin Invest. 2020; 130:2620–29. 10.1172/JCI137244 32217835 PMC7190990

[20] Klimek L, Jutel M, Akdis C, Bousquet J, Akdis M, Bachert C, Agache I, Ansotegui I, Bedbrook A, Bosnic-Anticevich S, Canonica GW, Chivato T, Cruz AA, et al, and ARIA-MASK Study Group. Handling of allergen immunotherapy in the COVID-19 pandemic: an ARIA-EAACI statement. Allergy. 2020; 75:1546–54. 10.1111/all.14336 32329930 PMC7264744

[21] Liu S, Zhi Y, Ying S. COVID-19 and asthma: reflection during the pandemic. Clin Rev Allergy Immunol. 2020; 59:78–88. 10.1007/s12016-020-08797-3 32468411 PMC8830198

[22] Gill MA, Liu AH, Calatroni A, Krouse RZ, Shao B, Schiltz A, Gern JE, Togias A, Busse WW. Enhanced plasmacytoid dendritic cell antiviral responses after omalizumab. J Allergy Clin Immunol. 2018; 141:1735–43.e9. 10.1016/j.jaci.2017.07.035 28870461 PMC6013066

