# Supplementary material for: Dynamic monitoring of immune function indexes in COVID-19 patients
Source: Aging (Albany NY). 2020 Dec 23;12(24):24596–603. doi: 10.18632/aging.202362 (PMC7803549; doi:10.18632/aging.202362)
Supplement: Supplementary Tables [file aging-12-202362-s001.docx]

**Supplementary Table 1. Clinical laboratory results of patients with 2019-nCoV pneumonia.**

| **Laboratory findings** | Normal range | case 1 | | case 2 | | case 3 | | case 4 | | case 5 | | case 6 | | case 7 | | | case 8 | | | case 9 | | |  |
| --- | --- | --- | --- | --- | --- | --- | --- | --- | --- | --- | --- | --- | --- | --- | --- | --- | --- | --- | --- | --- | --- | --- | --- |
| **Blood routine** |  | Before Treatment | After Treatment | Before Treatment | After Treatment | Before Treatment | After Treatment | Before Treatment | After Treatment | Before Treatment | After Treatment | Before Treatment | After Treatment | | Before Treatment | After Treatment | | Before Treatment | After Treatment | | Before Treatment | After Treatment | |
| leucocyte (×10⁹ per L) | 3.5-9.5 | 2.16 | 3.64 | 5.07 | 5.72 | 5.17 | 7.7 | 4.84 | 6.05 | 2.9 | 4.87 | 4.9 | 5.79 | | 6.04 | 5.9 | | 4.71 | 4.91 | | 2.67 | 4.74 | |
| neutrophil (%) | 40-75 | 84.7 | 62.4 | 78.2 | 63.9 | 68.4 | 62.3 | 59.3 | 44.5 | 65.5 | 53 | 61.4 | 63.8 | | 46.8 | 37.7 | | 59.3 | 53.6 | | 75.6 | 60.2 | |
| lymphocyte (%) | 20-50 | 10.3 | 22.8 | 10.7 | 22 | 24.6 | 29.4 | 28.6 | 43.8 | 26.9 | 31.4 | 29.4 | 29.5 | | 46 | 36.7 | | 24.8 | 37.1 | | 15.6 | 30.6 | |
| **Blood biochemistry** |  |  |  |  |  |  |  |  |  |  |  |  |  | |  |  | |  |  | |  |  | |
| Total protein | 65-85 | 62.9 | 73.24 | 58.64 | 61.56 | 63.1 | 66.9 | 65.6 | 73.5 | 66.4 | 65.8 | 62.3 | 65.41 | | 52.3 | **62.3** | | 74.88 | 69.7 | | 63.45 | 69.64 | |
| albumin (g/L) | 40-55 | 37.6 | 39.8 | 29.6 | 32.8 | 37 | 39.3 | 39.9 | 44.1 | 39.3 | 42.5 | 39.5 | 40.7 | | 39.2 | 40.1 | | 44.7 | 42.3 | | 36.1 | 39.3 | |
| ALT (IU/L) | 7-40 | 21 | 14 | 68 | 51 | 23 | 17 | 37 | 25 | 22 | 37 | 12 | 12 | | 11 | 13 | | 18 | 15 | | 17 | 12 | |
| AST (IU/L) | 13-35 | 36 | 18 | 97 | 34 | 34 | 17 | 32 | 18 | 17 | 14 | 22 | 18 | | 19 | 18 | | 44 | 41 | | 21 | 19 | |
| Prealbumin(mg/L) | 180-400 | 123 | 278 | 62 | 229 | 141 | 249 | 137 | 245 | 220 | 341 | 235 | 333 | | 125 | 217 | | 152 | 249 | | 144 | 249 | |
| LDH(IU/L) | 120-250 | 238 | 128 | 308 | 165 | 300 | 152 | 278 | 178 | 210 | 133 | 264 | 177 | | 262 | 156 | | 764 | 195 | | 278 | 247 | |
| HBDH(IU/L) | 72-182 | 223 | 123 | 255 | 139 | 252 | 127 | 216 | 146 | 187 | 117 | 204 | 152 | | 208 | 144 | | 502 | 154 | | 241 | 181 | |
| **Infection-associated** |  |  |  |  |  |  |  |  |  |  |  |  |  | |  |  | |  |  | |  |  | |
| C-reactive protein | 0-5 | 15.18 | 0.5 | 106.2 | 3.4 | 39.1 | 5.03 | 41.9 | 1.4 | 12.1 | 0.59 | 8.88 | 4.7 | | 20.54 | 3.6 | | 4.1 | 0.3 | | 0.67 | 0.01 | |
| **immunoglobulin** |  |  |  |  |  |  |  |  |  |  |  |  |  | |  |  | |  |  | |  |  | |
| IgG(mg/L) | 7-16 | 12.3 | 19.6 | 9.7 | 12 | 8.1 | 10.1 | 5.6 | 10.1 | 7.4 | 12.9 | 4.3 | 10 | | 4.7 | 9.8 | | 6.7 | 9.1 | | 4.2 | 9.1 | |
| IgM(mg/L) | 0.4-2.8 | 0.86 | 1.1 | 0.97 | 1.58 | 0.93 | 1.46 | 0.41 | 0.55 | 0.39 | 0.75 | 0.84 | 0.66 | | 0.76 | 1.16 | | 0.64 | 0.75 | | 0.47 | 0.75 | |
| IgA(mg/L) | 0.7-5 | 1.69 | 1.9 | 1.12 | 1.63 | 1.4 | 1.53 | 0 | 1.58 | 0.75 | 2.41 | 1.13 | 1.71 | | 0.87 | 1.5 | | 1.02 | 1.47 | | 0.65 | 1.47 | |
| IgE(mg/L) | 0-100 | 505 | 625 | 123 | 228 | 142 | 163 | 104 | 201 | 184 | 321 | 107 | 121 | | 79 | 154 | | 126 | 244 | | 201 | 244 | |
| **T cell subsets** |  |  |  |  |  |  |  |  |  |  |  |  |  | |  |  | |  |  | |  |  | |
| CD4(/μL） | 538-907 | 140 | 212 | 234 | 448 | 604 | 1316 | 395 | 424 | 424 | 525 | 419 | 575 | | 612 | 726 | | 471 | 543 | | 156 | 449 | |
| CD8(/μL） | 180-694 | 54 | 134 | 69 | 160 | 132 | 344 | 289 | 383 | 208 | 290 | 129 | 201 | | 365 | 598 | | 218 | 243 | | 114 | 363 | |
| CD4/CD8 | 0.89-2.01 | 2.59 | 1.58 | 3.37 | 2.8 | 4.57 | 3.83 | 1.37 | 1.11 | 2.04 | 1.81 | 3.26 | 2.86 | | 1.68 | 1.21 | | 2.16 | 2.23 | | 1.38 | 1.24 | |
| SARS-COV-2IgM(mg/L) | 0-1 | 0.5 | 0.757 | 0.98 | 4.243 | 1.24 | 3.965 | 0.45 | 5.70 | 0.51 | 0.84 | 0.68 | 1.52 | | 0.47 | 1.3025 | | 0.891 | 1.84 | | 1.456 | 2.014 | |
| SARS-COV-2IgG(mg/L) | 0-1 | 2.579 | 15.853 | 2.36 | 9.301 | 1.02 | 4.24 | 1.24 | 6.77 | 0.76 | 4.52 | 0.89 | 7.88 | | 0.56 | 0.89 | | 1.325 | 2.93 | | 3.697 | 10.016 | |
